# Rift Valley fever vector diversity and impact of meteorological and environmental factors on *Culex pipiens* dynamics in the Okavango Delta, Botswana

**DOI:** 10.1186/s13071-016-1712-1

**Published:** 2016-08-08

**Authors:** Hammami Pachka, Tran Annelise, Kemp Alan, Tshikae Power, Kgori Patrick, Chevalier Véronique, Paweska Janusz, Jori Ferran

**Affiliations:** 1 UPR AGIRs, F-34398, CIRAD, Montpellier, France; 2 Department of Zoology and Entomology, University of Pretoria, Pretoria, South Africa; 3 UMR CMAEE, F-34398, CIRAD, Montpellier, France; 4 UMR TETIS, F-34398, CIRAD, Montpellier, France; 5 Special Pathogens Unit, NICD, Johannesburg, South Africa; 6 Department of Veterinary Services, Ministry of Agriculture, Gaborone, Botswana; 7 Department of Animal Science and Production, Botswana College of Agriculture, Private bag 0037, Gaborone, Botswana

**Keywords:** Population dynamics modeling, Okavango Delta, Climatic factors, Flooding, Mosquito, *Culex pipiens*, Vector, Rift Valley fever, Remote sensing, Botswana

## Abstract

**Background:**

In Northern Botswana, rural communities, livestock, wildlife and large numbers of mosquitoes cohabitate around permanent waters of the Okavango Delta. As in other regions of sub-Saharan Africa, Rift Valley Fever (RVF) virus is known to circulate in that area among wild and domestic animals. However, the diversity and composition of potential RVF mosquito vectors in that area are unknown as well as the climatic and ecological drivers susceptible to affect their population dynamics.

**Methods:**

Using net traps baited with carbon dioxide, monthly mosquito catches were implemented over four sites surrounding cattle corrals at the northwestern border of the Okavango Delta between 2011 and 2012. The collected mosquito species were identified and analysed for the presence of RVF virus by molecular methods. In addition, a mechanistic model was developed to assess the qualitative influence of meteorological and environmental factors such as temperature, rainfall and flooding levels, on the population dynamics of the most abundant species detected (*Culex pipiens*).

**Results:**

More than 25,000 mosquitoes from 32 different species were captured with an overabundance of *Cx. pipiens* (69,39 %), followed by *Mansonia uniformis* (20,67 %) and a very low detection of *Aedes* spp. (0.51 %). No RVF virus was detected in our mosquito pooled samples. The model fitted well the *Cx. pipiens* catching results (ρ = 0.94, *P* = 0.017). The spatial distribution of its abundance was well represented when using local rainfall and flooding measures (ρ = 1, *P* = 0.083). The global population dynamics were mainly influenced by temperature, but both rainfall and flooding presented a significant influence. The best and worst suitable periods for mosquito abundance were around March to May and June to October, respectively.

**Conclusions:**

Our study provides the first available data on the presence of potential RVF vectors that could contribute to the maintenance and dissemination of RVF virus in the Okavango Delta. Our model allowed us to understand the dynamics of *Cx. pipiens*, the most abundant vector identified in this area. Potential predictions of peaks in abundance of this vector could allow the identification of the most suitable periods for disease occurrence and provide recommendations for vectorial and disease surveillance and control strategies.

**Electronic supplementary material:**

The online version of this article (doi:10.1186/s13071-016-1712-1) contains supplementary material, which is available to authorized users.

## Background

Mosquitoes are a major source of nuisance throughout the world, particularly because of the abundance of pathogens transmitted by their bites and their implications for animal and human health [1, 2]. Rift Valley fever virus (RVFV), an arbovirus belonging to the genus *Phlebovirus*, family Bunyaviridae, is one such pathogen. Transmitted to vertebrates by mosquitoes, mainly of the genera *Culex* and *Aedes*, or direct contact with viraemic animal products, it is responsible for Rift Valley fever (RVF), an acute disease considered as a significant global threat to both humans and animals [3, 4]. The virus has caused epizootics and human epidemics throughout Africa, the Arabian Peninsula and several islands of the Indian Ocean [5]. In ruminant livestock, especially sheep and cattle, the disease is characterized by high abortion and mortality rates (100 % in neonatal animals and from 10 % to 20 % among adult animals [6–8]). Human infections are characterized by blurred vision, retinal lesions, headache, loss of memory, lethargy, myalgia, fever and haemorrhages, although a minority (approximately 1 %) of the patients can develop complications such as encephalitis, ocular disease, retinitis or fatal haemorrhagic fever [9]. RVF, which significantly affects the health of animals and/or humans, induces a very heavy economic impact in the societies where it is present, and particularly in developing countries of tropical and sub-tropical areas [10]. In Botswana, as in other regions of sub-Saharan Africa, RVF virus is suspected to circulate among animals without reports of clinical outbreaks [11]. Indeed, RVF is often ignored and under reported in Botswana because clinical cases are not monitored and detected. To prevent and control RVF, effective knowledge about RVF risk areas and risk periods is required. As a mosquito-borne disease, RVF distribution in space and time is closely associated to the geographical distribution and dynamics of its vectors and hosts, which are themselves affected by climatic and landscape features [12–15]. Indeed, the ecology of vectors is highly dependent on environmental conditions. Each mosquito species requires specific environmental conditions to develop and survive, such as water availability to lay eggs, optimal temperature for aquatic stage development, limited wind (to facilitate host-seeking or breeding-site-seeking behaviour), or specific vegetation for some species [16].

The waters of the Okavango Delta allow the establishment of various mosquito species, such as *Culex* spp. breeding in the margins of permanent water bodies or *Aedes* spp. breeding in the marginal pools created by fluctuation of waters due to flooding. To date, in this region, no outbreaks of RVF have ever been reported. However, substantial RVF outbreaks have occurred in neighbouring countries (South Africa, Namibia, Zambia) [17] and serological investigations performed close to the time of this study provided evidence for the first time of RVFV antibody circulation among wild and domestic bovids in northern Botswana [11]. Consequently, the present study was structured around two main objectives: (i) Identifying the main potential vectors of RVFV present in the Okavango Delta; and (ii) Adapting and using a climate driven abundance model [18] under different biological scenarios to appreciate the potential influence of climatic and environmental factors, such as temperature, rainfall and water availability, on *Culex pipiens* populations, the main RVF mosquito vector species present at the livestock-wildlife interface in the Okavango Delta. It is commonly accepted that temperature is the main driver of transition rates between eggs, pupae, nymphs, and adult stages, and of mortality in mosquitoes [19]. This work specifically assessed the importance of the water availability and the respective roles of flood and rainfall. Therefore, we tested all possible combinations of these two ecological variables: temperature and water availability.

## Methods

### Study area

**Fig. 1:**
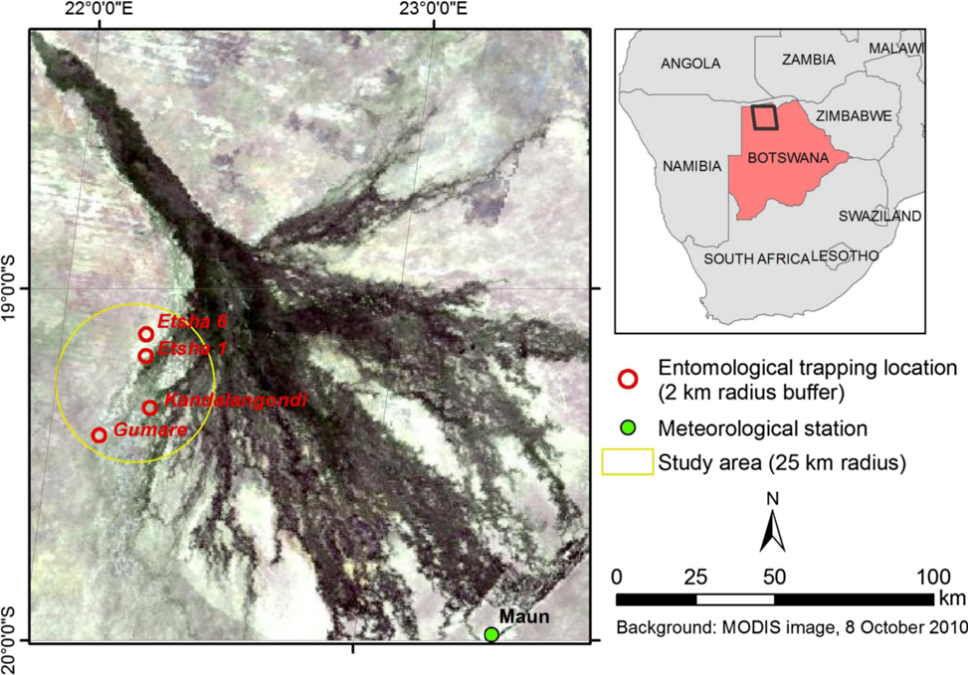
Map of the study area in north-western region of the Okavango Delta

The Okavango River basin, a unique wetland environment shared by Botswana, Angola and Namibia, supports a fragile and extremely complex ecosystem [20]. The study was carried out at the western border of the Delta located in semi-arid north-west Botswana, an interface between wetland and dry-land, precisely situated between 22°08′ and 22°18′E, and 19°07′ and 19°26′S (Fig. 1).

**Fig. 2:**
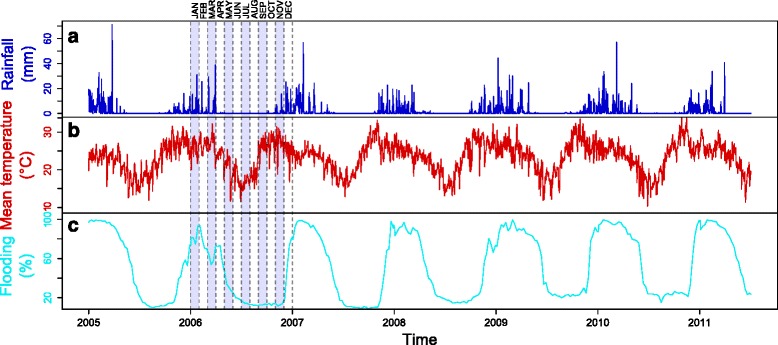
Meteorological and environmental data in the study area, Okavango Delta, 2005–2012. **a** Rainfall. **b** Mean daily temperature. **c** Proportion of flooded area dynamics over time

The hydrological system of the Delta is very complex and dominated by annual flood from the Okavango River. With the rivers that rise in the well-watered Angolan highlands and through Namibia, ‘disappearing’ into the sands of the Kalahari Desert and the “thirsty” atmosphere above the Okavango Delta, the whole Okavango system is endoreic in nature. Most rainfall occurs as summer downpours between October and March (Fig. 2a) ranging between 1,300 mm in parts of the catchment furthest to the north-west and steadily declining to 450 mm in the lowest reaches of the Delta [21]. The dry season, between April and November, is characterized by the almost total absence of rains. Maximum temperatures get warmer from September-October, soaring up to 40 °C, and dropping considerably towards the end of November or in early December (Fig. 2b). Nevertheless, until the end of February or early March, summer temperatures stay relatively warm, ranging from 38 °C during the day to 20 °C at night. During the dry winter season (April to September), day temperatures usually stand around 25 °C and evening temperatures can be as low as 2 °C and even go below freezing. The flood events are primarily caused by a flood wave induced by the high rainfall events happening in the northern catchment which lies entirely in the Angolan part of the Okavango River basin. During these events, depending on the size of the flood, the extent of the inundated area increases from 5,000 km^2^ to 6,000-12,000 km^2^ [22]. In addition, the flooding dynamics depends on the highly seasonal rainfall to which the Delta area is exposed between October and March (Fig. 2a,c). The spatial variability in available water is presented later in this paper.

Few people live permanently in the area of the inner Delta and the remote areas of western and northern Ngamiland (district located around the Delta in northwestern Botswana). Most of the existing settlements are very small, with a high proportion of the population clustered in a handful of larger villages and in the district capital of Maun (Fig. 1). In total, the Ngamiland District population is composed of about 150,000 persons [23]. Most land is used for communal farming, private ranching, wildlife conservation and tourism. The extremely rich biodiversity of the Delta and high abundance of large mammals results in the great diversity of habitats ranging from different kinds of drylands to various wetlands and the obvious availability of a perennial supply of water. The conditions are poorly suited to agriculture and ranching because soils have low fertility, rainfall varies too much to produce good yields and pastures cannot support large numbers of livestock, all the more so because livestock diseases and crop pests limit production. Most livestock is kept on communal or tribal land, and adjacent to the buffalo veterinary fence erected to limit the spread of disease between wildlife and livestock. Cattle and goats are the most abundant stock, with smaller numbers of sheep and donkeys [20]. Cattle numbers in the western part of the Delta, where this research was implemented, are estimated to be approximately 20,000 head and their density (32 cattle heads/km^2^) is the highest among the wildlife/livestock interface of Northern Botswana [24].

### Data

#### Mosquito sampling

Sampling sites to collect mosquitoes were selected on the basis of the detection of RVFV antibodies in livestock during a preliminary serological survey undertaken in buffalo and livestock in April 2010. The sampling design is described in Jori et al*.* [11], see summary of methods and results in Additional file 1. This survey enabled us to identify, in the study area, cattle corrals (isolated farms) with significant seroprevalence rates for antibodies against RVFV (Table 1). In our study, those seroprevalence rates were used as a proxy of mosquito abundance. Sampling was performed by disposing the traps between 25 and 50 m from cattle corrals located in the areas named Etsha 1, Etsha 6, Kandalangondi and Gumare (Fig. 1). Despite measures of meteorological parameters on the capture sites were not taken, climate, topography and landscape in the 25 km^2^ area seemed quite homogeneous and we assumed that the four mosquito capture sites had very similar environmental and ecological conditions. For instance, the altitude in the four sites were very similar: Etsha 1 is located 973 m above sea level (asl), Etsha 6 is 974 m asl, Kandalangondi is 966 m asl and Gumare is 964 m asl.

**Table 1:** Seroprevalence of IgG antibodies detected in the five different crush pens^b^ located on the Western boundary of the Okavango Delta

	Number of positives^a^/Total number tested	Observed crush pen prevalence (%)
Gumare	6/50	12
Kandalangondi	11/50	22
Etsha 1	37/50	74
Etsha 6	15/50	30
Total	69/200	34.5 ± 27.3

Sampling, method and analysis are presented in Additional file 1

^a^Dilution higher than 1/10

^b^Crush pens or diptanks are crushing devices where livestock herds from different owners sharing the same grazing lands in southern Africa congregate regularly (approximately once a month) to receive vaccination and be monitored against notifiable animal diseases such as foot-and-mouth disease

Trapping episodes took place from sunset (between 15:30 and 18:00) to sunrise (6:00 to 8:00). The traps consisted of a rectangular “tent” of fine netting spread over 4 aluminum corner poles and had dimensions of 2 m long × 1.5 m wide × 1.6 m high. The trapping method is based on the attraction of mosquitoes to carbon dioxide and RVFV hosts proximity [25]. Dry ice was used as a source of carbon dioxide to bait mosquitoes just before sunset in order to collect the mainly crepuscular/nocturnal vector species [26]. The netting was rolled up to approximately 15 cm from the ground to allow mosquito entry and left overnight. The tent traps were cleared of mosquitoes just before sunrise, by entering the trap, rolling the sides down to ground level and collecting the mosquitoes with a mechanical aspirator. Mosquitoes were transferred to small cages for transport to the base station, where they were killed by freezing for 20 min at -20 °C, sorted according to genus and stored in 1.8 ml cryotubes in lots of up to 200 per tube. The sorted mosquitoes were then frozen at -20 °C or lower for preservation and transported to the laboratory on dry ice for species identification and virological testing. The sampling extended from April 2011 to March 2012 during the summer rainfall season excluding the winter months from May to September. During the sampling period, in each location one night per month, 6 traps were set; 4 traps were disposed at the 4 cardinal points surrounding the cattle corral and the 2 remaining traps were disposed around the cardinal point on the closest side from the Delta. One trapping episode failed because of heavy rain during the night. In total, 23 trapping episodes were undertaken under good meteorological conditions (Table 2).

**Table 2:** Global mosquito dynamics. Trapping summary for the four sites with the date of each trapping episode, the number of tubes collected (Nb) and the number of collected *Culex pipiens* among all mosquitoes collected by trapping session. The date format used was dd.mm.yy

Site	Etsha 1			Kandalangondi			Gumare			Etsha 6		
	Date	Nb	*Cx. pipiens*	Date	Nb	*Cx. pipiens*	Date	Nb	*Cx. pipiens*	Date	Nb	*Cx. pipiens*
	08.04.11	34	1,738/3,849	09.04.11	19	1,394/2,418	29.04.11	11	50/154	30.04.11	10	619/714
	28.09.11	6	2/39	27.09.11	5	2/205	30.09.11	1	0/1	29.09.11	3	0/4
	22.11.11	6	2/11	21.11.11	5	0/11	24.11.11	4	3/4	23.11.11	3	0/5
	17.12.11	14	278/757	16.12.11	3	12/113	19.12.11	5	8/40	18.12.11	6	1/16
	25.01.12	7	120/307	24.01.12	2	0/6	27.01.12	4	2/22	26.01.12	HR	
	07.03.12	46	3,289/8,315	05.03.12	38	10,396/11,509	08.03.12	6	7/23	09.03.12	6	0/503
Total		113	5431/13,376		72	11,809/14,279		31	964/1,271		28	63/222

*Abbreviations*: *HR* heavy rainfall prevented the trapping session; *Nb* number of tubes collected

Identification of the trapped mosquito specimens was performed according to the keys and descriptions of Jupp [27], Edwards [28], Gillies & de Meillon [29] and Gillies & Coetzee [30]. To test the presence of RVFV in vectors, a total of 18,259 mosquitoes divided in mosquito pools were processed to obtain supernatant fluid as described by Jupp et al*.* [31], which was inoculated in infant mice (NHLS Animal Ethics Clearance Certificate No. 124/11). Those mice were tested by RT-PCR. We concentrated on species known as vectors of RVFV [32–34].

#### Temperature

Daily temperature data were recorded by the Department of Meteorological Services of Botswana at Maun airport located at the Delta’s southern fringe, which is the closest meteorological station to the study area (Fig. 1) and has been recording daily climatic data since 1921.

#### Rainfall

Daily rainfall data were estimated from satellite Tropical Rainfall Measuring Mission (TRMM) at 0.25° spatial resolution, downloaded from NASA’s Goddard Earth Sciences Data and Information Services Center from 2005 to 2012 [35].

#### Mapping flooding extent

To assess the hydrological dynamics, we used inundation extent measures derived from Moderate Resolution Imaging Spectroradiometer (MODIS) satellite imagery. The daily proportion of flooded areas was computed around each trapping location (Etsha 1, Etsha 6, Gumare, Kandalangondi) within a 2 km radius and at a study area scale defined as the area around the centroid of the four sites within a 25 km buffer radius (Fig. 1). Computation was performed using Geographic Information System (GIS) functionalities following the method detailed in Additional file 2.

### Modeling mosquito population dynamics

#### Model design and analysis

A continuous time and stage structured model designed by Cailly et al*.* [18] to study mosquito population dynamics and applied in European wetlands was adapted in order to simulate the population dynamics of the main RVFV vector in the north-western region of the Okavango Delta.

**Fig. 3:**
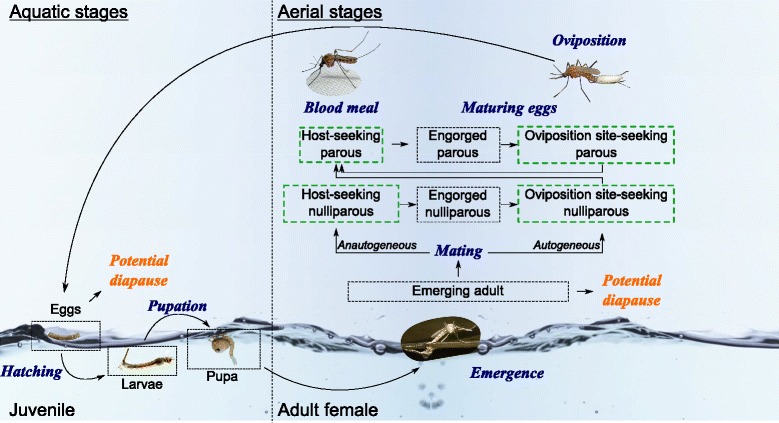
Diagram of the generic model of mosquito population dynamics based on life-cycle inspired from Cailly et al. [18], succession of stages (not italicized *black* text) and events (italicized *blue* text). Mosquito life-cycle contains a complete metamorphosis between aquatic juvenile stages drawn on the left of the dotted line and terrestrial adult stages on the right. As in Cailly et al*.* [18], females were divided into nulliparous (which have never laid eggs) and parous (which have laid eggs at least once). The green dotted box indicates the females which moved to seek a host or an oviposition site. *Culex* species enter diapause as young adults (top-right-arrow) while *Aedes* species enter diapause as egg (bottom-right-arrow)

The model considers the entire mosquito life-cycle (Fig. 3). It uses an *a priori* mechanistic mathematical description of all processes of mosquito population dynamics and a deterministic representation of the average behaviour of the population. The model includes meteorological and environmental conditions, such as water presence and temperature, and specific processes of mosquito life-cycle, such as diapause.

The model was run through seven consecutive years. The dynamics in year *n* + 1 explicitly depends on the dynamics in year *n* and on survival rates during the unfavourable seasons. As in Cailly et al*.* [18], ten different stages were considered: 3 juvenile aquatic stages (*E*, eggs; *L*, larvae; *P*, pupae), 1 emerging adult stage *A*_*em*_), 3 adult nulliparous female stages (*A*_*1h*_, *A*_*1g*_, *A*_*1o*_, and 3 adult parous female stages (*A*_*2h*_, *A*_*2g*_, *A*_*2o*_) (Fig. 3). Adults were subdivided regarding behaviours during the gonotrophic cycle, which could affect their survival (*h*, host-seeking; *g*, transition from engorged to gravid; *o*, oviposition site seeking). Individual transitions between stages were due to different demographic events such as egg mortality and hatching, larval mortality, pupation (moult of larvae to pupae), pupa mortality, adult emergence, adult mortality, adult engorgement, egg maturing or oviposition (Fig. 3). Density-dependent mortality is assumed at the larval and egg stages [16, 36]. The success of adult emergence is considered dependent and negatively correlated to pupal density [37]. Because of their inability to feed on blood excludes them from the pathogen transmission process, adult males were excluded from future computations.

To take on board the environmental influence and the seasonality of the mosquito life-cycle, Cailly et al*.* [18] based their model on two systems of ordinary differential equations (ODE): one for the favourable period, during which mosquitoes were active, and the other for the unfavourable period, during which, according to the temperature level and the length of the day, diapause occurred [19]. The diapause of *Culex pipiens* in southern Africa consists of a combination of winter die-off and survival of a portion of the adult females in refuges such as reed beds (natural wetland habitat widely occurring in the Okavango Delta region and characterized by a vegetation dominated by reeds). Larvae may also survive in the reed beds [38–40]. For *Culex* and *Anopheles* species, for which nulliparous adults were those surviving during the diapause, the ODE systems were detailed in Additional file 3.

#### Adaptation of the mosquito population model to the main RVFV vector species of Okavango Delta

Once the main species present in our study area were identified, we defined the model parameters using published information and expert opinion. Systematic literature reviews were performed in order to define the values of model parameters for the most abundant species, using words related to the life-cycle of mosquitoes, the name of the species and to this specific region in southern Africa. Knowledge gaps in the literature were addressed by organizing discussions with entomologists working in the region (Botswana, South Africa) in order to discuss the missing parameter values. According to local expert opinion and literature review, we defined the favourable period beginning on the 1st September and ending on the 30th June.

The model was used with daily temperature, flooding and rainfall as inputs to highlight the main meteorological and environmental drivers of mosquito population dynamics in the study area. A daily temperature dataset collected from Maun meteorological station was the same for the four study sites, assuming a low variability in temperature among the study sites because of the very low difference in altitude between sites (maximum 10 m) and with Maun (maximum 26 m). As we assumed, differences between the rainfall measured in Maun meteorological station and local rainfall in the study area, the latter was estimated from satellite estimates. Given the proximity between the four sites and the absence of relief, rainfall was considered homogeneous on the study area. Nevertheless, spatial variations in flooding were accounted for by the use of local satellite-derived datasets - flooding was estimated independently for each site (site-scale). We tested four scenarios based on different meteorological and environmental factor influence on the population dynamics of main RVF vector in Okavango Delta: *Scenario 1)* Temperature alone is driving the population dynamics; *Scenario 2)* A combination of temperature and rainfall is sufficient to forecast the population dynamics; *Scenario 3)* A combination of temperature and flooding is sufficient to forecast the population dynamics; and  *Scenario 4)* A combination of temperature, rainfall and flooding is required to forecast the population dynamics.

#### Initial conditions

Each run was initiated during the unfavourable season, when mosquito numbers were supposed to be the lowest. In our study area, this date corresponds with the month of July. Therefore, the model was initialized in this month with an initial population of 10^7^ emerging adults. The outputs of the model were the simulated daily numbers of individuals at each stage.

#### Model validation

To validate the model, we compared model outputs of simulated host-seeking adult abundance (*A*_*1h*_ + *A*_*2h*_) for the years 2011–2012 with field data. For each scenario, the degree of association between observed and simulated data at the time of trapping was assessed by calculating the Spearman correlation coefficient **ρ**. We used an aggregation of field data from the four sites and ran the scenarios 3 and 4 using flooding extents estimated at the study area scale. Then, in order to assess the ability of the model to predict the spatial variation of the abundance and still using the Spearman correlation coefficient **ρ**, we compared the simulated - from scenario 4 - and observed maximal abundance for each site independently. In order to do this, we used field data from each site independently of each other and compared them with abundances simulated independently for each site using site-scaled estimates of flooding extents (temperature and rainfall being assumed similar on the four sites).

#### Variance-based sensitivity analysis

We used a global sensitivity analysis, varying simultaneously all of the model parameters [41], to assess the dependence of the model to these different factors. The method tested the influence of the input variation on the normally distributed aggregated outputs. This analysis enabled us (i) to determine the key parameters involved in the biological dynamic system; and (ii) to adjust the functions together with the model.

We determined four aggregated outputs per year: (i) the maximal number of mosquito females (abundance peak); (ii) the proportion of host-seeking females per day, averaged in a time window of 20 days around the abundance peak (host-seeking rate); (iii) the parity rate, which is the proportion of parous females per day, averaged in a time window of 20 days around the abundance peak; and (iv) the date of the emergence of most mosquitoes from aquatic and immature to adult and aerial stages. The abundance peak enabled us to characterize the mosquito dynamics while the parity rate provided an estimation of the proportion of mosquitoes having already taken a blood meal, therefore, potentially infected - of course, the proportion of infected mosquitoes will usually be less than the proportion that have taken a blood meal and the proportion of infected mosquitoes that transmit will be less than the number infected.

We produced a data set where each parameter of the model varied independently by approximately 10 %, 25 % and 50 %. Simulations using this data set enabled us to estimate the contribution of each single parameter to a minimum of 90 % of the variance. Therefore, only the main factors or interactions accounting for these 90 % of the output variance were retained as key parameters for the model [42]. The contribution of the variation factors to the output variability was evaluated using a linear regression, with the aggregated output as response and the parameters as explanatory variables. Demographic simulations were performed using Scilab (version 5.4.1) [43], and statistical simulations and plotting were performed using R (version 3.1.2) [44], free and open-source software for numerical computation and graphics.

## Results

### Mosquito capture results

A total of 26,289 mosquitoes from 32 different species were identified (Table 3). Our trapping records revealed a large difference in the number of trapped mosquitoes. Indeed, while an important number of mosquitoes (> 24,900) was collected in the locations of Etsha 1 and Kandalangondi (11,927 and 13,044, respectively), a much lower number of mosquitoes (<1,500) was trapped in Etsha 6 and Gumare (1,102 and 216, respectively). The three main species representing respectively 69 %, 21 % and 4 % of the total number of identified mosquitoes were *Culex pipiens* with 18,267 individuals, *Mansonia* (*Mansonioides*) *uniformis* with 5,429 specimens and *Mansonia* (*Mansonioides*) *africana* with 1,140 individuals. It should be noticed that a very low number of *Aedes* (*Aedes*) was detected (4 species including 134 individuals) mostly represented by *Ae.* (*Neomelaniconion*) *mcintoshi* and *Ae.* (*Neomelaniconion*) *unidentatus*. Despite all these species being able to transmit RVFV [45, 46], no evidence of RVFV or other arboviruses was found in the mosquito pools inoculated to mice.

**Table 3:** Summary of the identified species of mosquito

Species	Number of identified specimens	%
*Culex* (*Culex*) *pipiens*	18,267	69.49
*Mansonia* (*Mansonioides*) *uniformis*	5,429	20.65
*Mansonia* (*Mansonioides*) *africana*	1,140	4.34
*Anopheles* (*Cellia*) *squamosus cydippus*	302	1.15
*Coquillettidia* (*Coquillettidia*) *fuscopennata*	266	1.01
*Culex* (*Culex*) *neavei*	230	0.87
*Anopheles* (*Cellia*) *pharoensis*	196	0.75
*Culex* (*Culex*) *poicilipes*	86	0.33
*Aedes* (*Neomelaniconion*) *mcintoshi*	85	0.32
*Anopheles* (*Cellia*) *argenteolobatus*	81	0.31
*Culex* (*Culex*) *univittatus*	54	0.21
*Aedes* (*Diceromyia*) *adersi*	45	0.17
*Culex* (*Eumelanomyia*) *horridus*	25	0.10
*Anopheles* (*Anopheles*) *tenebrosus*	19	0.07
*Coquillettidia* (*Coquillettidia*) *microannulata*	10	0.04
*Anopheles* (*Anopheles*) *ziemanni namibiensis*	8	0.03
*Anopheles* (*Cellia*) *arabiensis*	7	0.03
*Anopheles theileri*	6	0.02
*Anopheles* (*Anopheles*) *caliginosus*	5	0.02
*Culex* (*Culex*) *quinquefasciatus*	5	0.02
*Anopheles* (*Cellia*) *tchekedii*	4	0.02
*Aedes* (*Neomelaniconion*) *unidentatus*	3	0.01
*Anopheles* (*Anopheles*) *implexus*	3	0.01
*Anopheles* (*Cellia*) *pretoriensis*	3	0.01
*Coquillettidia*(*Coquillettidia*) *chrysosoma*	3	0.01
*Aedeomyia* (*Lepiothauma*) *furfurea*	1	0.00
*Aedeomyia* (*Lepiothauma*)*africana*	1	0.00
*Aedes* (*Aedimorphus*) *argenteopunctatus*	1	0.00
*Anopheles* (*Cellia*) *distinctus*	1	0.00
*Anopheles* (*Cellia*) *kingi*	1	0.00
*Coquillettidia* (*Coquillettidia*) *flavocincta*	1	0.00
*Culex* (*Culex*) *striatipes*	1	0.00

We decided to base the model on *Cx. pipiens*, because this species presented the strongest variation in abundance over time with 3,806 mosquitoes caught in April 2011, 4 in September 2011, 6 in November 2011, 301 in December 2011, 122 in January 2012 and 14,028 in March 2012 (Table 2) and it is also the one species in our collections most strongly associated with transmission of RVFV elsewhere [33, 47, 48].

### Estimation of environmental and meteorological environment

**Fig. 4:**
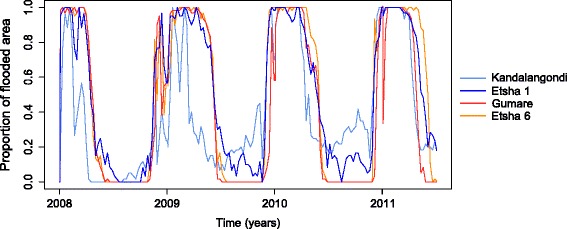
Proportion of flooded area dynamics over time in the four trapping sites, Okavango Delta, 2005–2012

The daily variations in rainfall, temperature and flooding on the global study area were presented in Fig. 2a, b, c, respectively. The estimated spatial variation of flooding illustrated in Fig. 4 showed more abundant water flock in Kandalangondi and Etsha 1 (dark and light blue lines) than in Gumare and Etsha 6 (red and orange lines) during the dry season.

### Definition of the parameters and functions of the model for *Cx. pipiens*

A list of 21 constant parameters related to the bio-ecology of *Cx. pipiens* in subtropical areas was computed from existing literature and expert opinion (Table 4). Our estimation of carrying capacity was based on the maximum density of individuals (larvae or pupae) and on the available breeding surface in a hectare (considering adult females lay their eggs only on the border of the water surface).

**Table 4:** Description of the different parameters used in the model

Parameter	Definition	Value	Reference
*β*_1_	Number of eggs laid by ovipositing nulliparous females (per female)	141	[37, 51, 67, 75]
^*β*^2	Number of eggs laid by ovipositing parous females (per female)	80	[37, 51, 67, 75]
_*k*_ *Lmin* _*k*_ *Lmax*	Standard environment carrying capacity for larvae (larvae ha^-1^)	8 × 10^6^ 1 × 10^8^	Expert opinion
*k*_*P min*_ *k*_*P max*_	Standard environment carrying capacity for pupae (pupae ha^-1^)	8 × 10^6^ 1 × 10^8^	Expert opinion
*σ*	Sex-ratio at the emergence	0.5	[16, 36]
*μ*_*E*_	Egg mortality rate (day^-1^)	0.0262	[37, 51, 67, 75]
^*μ*^ *L*	Minimum larva mortality rate (day^-1^)	0.0304	[37, 51, 67, 75]
^*μ*^ *P*	Minimum pupa mortality rate (day^-1^)	0.0735	[37, 51, 67, 75, 76]
^*μ*^ *em*	Mortality rate during adult emergence (day^-1^)	0.21845	[67, 77]
^*μ*^ *A*	Minimum adult mortality rate (day^-1^)	0.1	[77]
*μ*_*r min*_ *μ*_*r max*_	Adult mortality rate related to seeking behaviour (day^-1^)	0.18 0.59	[78]
^*γ*^ *Ah*	Transition rate from host-seeking to engorged adults (day^-1^)	0.885	[37, 51, 67, 75]
^*γ*^ *Ao*	Transition rate from engorged adult to oviposition site-seeking adults (day^-1^)	0.25	[67]
^*γ*^ *Aem*	Development rate of emerging adults (day^-1^)	0.25	[37, 51, 67, 75]
*T*_*E*_	Minimal temperature needed for egg development (°C)	10	[16]
*TDD*_*E*_	Total number of degree-day necessary for egg development (°C)	19.18	[16]
*T*_*Ag*_	Minimal temperature needed for development of engorged females (°C)	10	[37, 51, 67, 75]
*TDD*_*Ag*_	Total number of degree-days necessary for engorged females development (°C)	64.4	[37, 51, 67, 75]

“Expert opinion”: estimation from others species or areas [37]

According to bibliographic review and expert opinion on the bio-ecology of *Cx. pipiens*, we defined the impact of meteorological and environmental variables as follows: transitions between successive stages (*f*_X_) were all temperature-driven for the aquatic stages, as well as the duration of egg maturation (transition from engorged to gravid: *f*_*Ag*_) [38]. Moreover, all mortality rates were temperature-driven, except the egg mortality [37]. We integrated a relationship between adult mortality related to seeking behaviour (*mr*) and the environmental variables, considering that *mr* was inversely correlated with the water availability, indeed the farther mosquitoes need to pull away from the water to find a host, the higher is their probability of dying. We also considered that water availability impacts the environment’s carrying capacity of aquatic stages (*k*_*L*_ and *k*_*P*_), flooding increasing the number of breeding sites available. Definitions of the model functions for *Cx. pipiens* species were provided in Table 5.

**Table 5:** Functions describing *Cx. pipiens* life-cycle

Definition	Function	Reference
Egg hatching function (rate of egg reaching the following stage)	${f}_E(t)=\frac{T(t)-{T}_E}{TD{D}_E}$	[18, 79]
Larval development function (rate of larvae turning in pupae)	${f}_L(t)=\frac{0.021\times {e}^{0.162\times \left(T(t)-10\right)} - {e}^{0.162\times \left(T(t)-10\right)-\frac{35-T(t)}{5.007}}}{4}$	[18, 67]
Pupal development function (rate of pupae emerging)	${f}_L(t)=0.021\times {e}^{0.162\times \left(T(t)-10\right)} - {e}^{0.162\times \left(T(t)-10\right)-\frac{35-T(t)}{5.007}}$	[18, 67]
Rate of adults becoming gravid	${f}_{Ag}(t) = \frac{T(t)-{T}_{Ag}}{TD{D}_{Ag}}$	[18, 79]
Larval mortality rate	${m}_L(t) = {e}^{\frac{-T(t)}{2}}+{\mu}_L$	[18, 80]
Pupal mortality rate	${m}_P(t) = {e}^{\frac{-T(t)}{2}}+{\mu}_P$	[18, 80]
Daily adult mortality rate	*m*_*A*_(t) = -0.005941 + 0.002965 × T(t)	[18, 67, 80]
Additional adult mortality rate related to the seeking behaviour	*m*_*r*_(*t*) = *μ*_*r max*_ − *W*(*t*) × (*μ*_*r max*_ − *μ*_*r min*_)	BK
Daily environment carrying capacity for larvae	*κ*_*L*_(*t*) = *κ*_*L min*_ + *W*(*t*) × (*κ*_*L max*_ − *κ*_*L min*_)	BK
Daily environment carrying capacity for pupae	*κ*_*P*_(*t*) = *κ*_*P min*_ + *W*(*t*) × (*κ*_*P max*_ − *κ*_*P min*_)	BK

*BK* to our best knowledge, *T* daily average temperature, *W* daily average water presence:$$ W(t) = \left\{\begin{array}{c}\hfill 1\hfill \\ {}\hfill R(t)\hfill \\ {}\hfill F(t)\hfill \\ {}\hfill \frac{R(t)+F(t)}{2}\hfill \end{array}\ \right.\begin{array}{c}\hfill \mathrm{in}\ \mathrm{scenario}\ 1,\hfill \\ {}\hfill \mathrm{in}\ \mathrm{scenario}\ 2,\hfill \\ {}\hfill \mathrm{in}\ \mathrm{scenario}\ 3,\hfill \\ {}\hfill \hfill \\ {}\hfill\ \mathrm{in}\ \mathrm{scenario}\ 4,\ \hfill \end{array} $$

With R: 8 days cumulated rainfall; F: daily flooding (normalized values)

All of these functions were modified according to the four hypotheses on the meteorological and environmental factors suspected to influence on the population dynamics of *Cx. pipiens* in Okavango Delta. Those functions can take into account daily temperature (*T*) (all scenarios) and different rates of water presence (*W*), including either 8 days cumulated rainfall (Scenario 2), or the flooding area proportion (Scenario 3), or the mean of both (Scenario 4).

### Simulation and validation

**Fig. 5:**
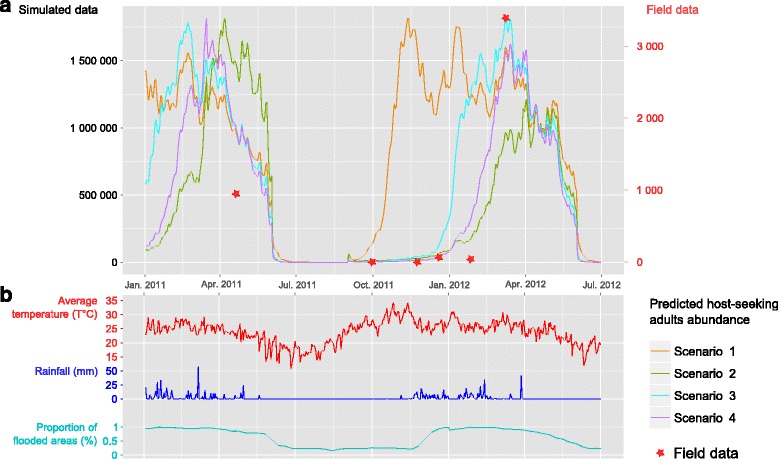
Confrontation between aggregated field data (*red* stars), simulations (*coloured lines* on the upper graphic) under the four scenarios (**a**) and environmental variations (**b**). Scenario 1: Temperature used as unique input to describe the *Culex pipiens* population dynamics. Scenario 2: Combination of temperature and rainfall used as inputs to describe the *Culex pipiens* population dynamics. Scenario 3: Combination of temperature and flooding used as inputs to describe the *Culex pipiens* population dynamics. Scenario 4: Combination of temperature, rainfall and flooding used as inputs to describe the *Culex pipiens* population dynamics

The model was run for a seven-year period (from the beginning of the favourable period of 2005 to the one of 2012). Figure 5 shows the host-seeking adult mosquitoes abundance predicted by the four scenarios plotted with a temporal superimposition of trapping records (mean on the four sites per month), simulated abundances and climatic and environmental variations. The simulated population dynamics present a strong temporal variability (Fig. 5a) following environmental and meteorological temporal variation (Fig. 5b). According to the used scenario, the starting date of adult emergence moves back or forward in the year: October in scenario 1, January in scenario 3, February in scenarios 2 and 4; nevertheless the date of the peak of mosquitoes seemed to be subject to variation.

#### Scenario 1 - temperature alone has an effect on mosquito dynamics

The simulated number of host-seeking mosquitoes under the hypothesis of temperature as the only driver of mosquito dynamics (orange line in Fig. 5) increased as soon as the favourable period begun according to the increase of temperature in November. The global mosquito predicted abundance was poorly correlated with trapping collection results. The Spearman correlation coefficient (ρ) was 0.26 (*P* = 0.658).

In the same way, tests of comparison applied to each site separately showed low correlations: ρ = 0.06 for Etsha 1 (*P* = 0.913), ρ = -0.20 for Kandalangondi (*P* = 0.700), ρ = -0.12 for Gumare (*P* = 0.827), ρ = 0.15 for Etsha 6 (*P* = 0.805).

#### Scenario 2 - combined effects of temperature and rainfall have a significant influence on mosquito dynamics 

The host-seeking headcount simulated using rainfall amounts as a second driver of the *Culex pipiens* dynamics in addition to temperature (green line in Fig. 5) increased several months after the beginning of the favourable period (April) and did not reach a peak as high as the other simulations. Simulations were significantly more correlated with the global *Cx. pipiens* population dynamics on the four sites. The Spearman correlation coefficient (ρ) was 0.89 (*P* = 0.033).

The Spearman’s tests applied to each site separately showed distinct patterns. The correlation for the sites located close to the Okavango River, Etsha 1 (ρ = 0.87, *P* = 0.024) and Etsha 6 (ρ = 0.97, *P* = 0.005), was higher than for the two further sites: Kandalangondi (ρ = 0.61, *P* = 0.200) and Gumare (ρ = 0.38, *P* = 0.461).

#### Scenario 3 - combined effects of temperature and flooding influence mosquito dynamics 

Scenario 3 used the proportion of flooded surface estimated in the smallest area including the four sites (study area scale) as the second driver of the mosquito dynamics, in addition to temperature. The simulated number of host-seeking mosquitoes (cyan line in Fig. 5) increased a few months after the beginning of the favourable period (February-March) and quickly reached a high quantity. Simulations well fitted to the field data: the Spearman correlation coefficient was equal to 0.83 (*P* = 0.058).

The Spearman’s test applied to each site separately was quite close to those computed from scenario 2 simulations. The rho coefficient were very significant for the two sites with trapping locations closer to the Okavango River and more impacted by flooding: Etsha 1 (ρ = 0.93, *P* = 0.008) and Etsha 6 (ρ = 0.054). The correlations with southern sites: Kandalangondi (ρ = 0.46, *P* = 0.354) and Gumare (ρ = 0.12, *P* = 0.827) farther from the river were not significant.

#### Scenario 4 - temperature, rainfall and flooding, have a significant effect on mosquito dynamics

The number of host-seeking mosquitoes simulated according to the last scenario involving proportion of the flooded surface on the Okavango Delta, rainfall and temperature as three significant drivers of the mosquito dynamics (purple line in Fig. 5) presented an intermediate pattern between scenarios 2 and 3. Spearman’s coefficient between predicted values and global field data being the highest (ρ = 0.94, *P* = 0.017) testified a strong correlation.

Considering each site separately, using temperature, flooded area proportion at site-scale and rainfall as drivers of the population dynamics, the global correlation between predictions and field data produces the strongest coefficients of determination for Etsha 1: ρ = 0.87 (*P* = 0.024) and Etsha 6: ρ = 0.97 (*P* = 0.005). The correlations with trapping records from Kandalangondi: ρ = 0.67 (*P* = 0.148) and Gumare: ρ = 0.38 (*P* = 0.461) were also relatively high but still less significant than for the other sites.

### Spatial dynamics

**Fig. 6:**
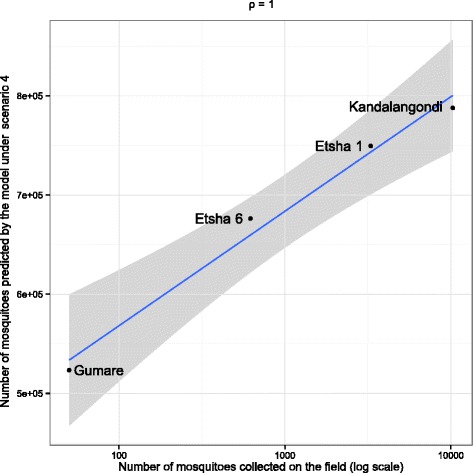
Separate confrontation between abundance of mosquitoes collected on each field mosquito capture site and the abundance expected by site-scale simulation under scenario 4 with the Spearman’s rho coefficient

Simulations using the proportion of flooded surface estimated at the local scale (site-scale) in scenario 4 seemed able to qualitatively predict locations where mosquitoes are going to be more abundant than somewhere else. Figure 6 illustrates the correlation between the maximum number of mosquitoes collected in each of the four study sites, and the maximum number of mosquitoes as predicted by the model, using a bi-dimensionnal representation of the data. Using scenario 4 simulations, the Spearman’s correlation between maximal abundance of collected mosquitoes and the predicted number of mosquitoes over one year was extremely significant (ρ = 1, *P* = 0.083; Fig. 6). The model was able to accurately replicate the spatial variation of the number of mosquitoes between the different sites.

### Sensitivity analysis

**Fig. 7:**
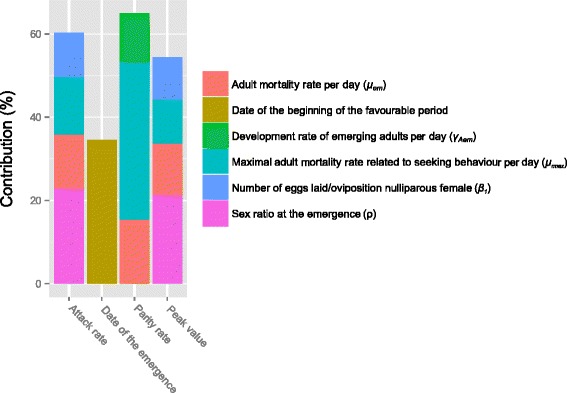
Key parameters contributing to aggregated outputs variance in scenario 4. Adult mortality rates, development rate of emerging adult and sex ratio at the emergence, number of eggs laid per nulliparous female and beginning of the favourable period significantly affect the outputs of our model

The sensitivity analysis computed on the aggregated outputs (the abundance peak value, the host-seeking rate, the parity rate and the date of the peak of emergence of adults) of the model run under scenario 4 allowed us to identify six key parameters contributing to the aggregated output variance: (i) the adult mortality rate at the emergence; (ii) the date of the beginning of the favourable period; (iii) the development rate of emerging adults per day; (iv) the maximum adult mortality rate related to seeking behaviour; (v) the number of eggs laid by oviposition of nulliparous female; and (vi) the sex-ratio at the emergence (Fig. 7). The peak value and the host-seeking rate were affected by the same parameters. The date of the emergence was related to the beginning of the favourable period. The parity rate was mostly driven by adult mortalities related to the host seeking behaviour.

## Discussion

An important observation relative to the trapping results was the overabundance of *Cx. pipiens* (> 8,000) compared to the other species (> 60 %), and especially, compared to the small number of *Aedes* spp. (< 150; < 1 %) collected. The overabundance of *Cx. pipiens* and the very low numbers of *Aedes* spp. could involve different processes. A possible explanation was that the Okavango Delta, with permanent water all year round, would be unfavourable to *Aedes* spp. which lay its eggs on damp soil and requires an event of desiccation for the eggs to hatch. Finally, *Aedes* eggs hatching irregularly triggered by flooding events [49–51], a single trapping session per month could have failed to capture this species of mosquitoes at the moment when these species were more abundant. The implementation of more frequent trapping sessions over the year, without a break during the dry season may enable this hypothesis to be tested. Similar observations regarding disparity between *Culex* spp. and *Aedes* spp. were made in other delta areas such as the Senegal River [52]. Another hypothesis was that the lack of field data relative to the winter months, between April and September, could have hidden a higher presence of *Aedes* spp. (Alan Kemp personal communication, [26, 53, 54]).

A strong spatial variability in catches was observed between the four trapping sites (Table 2), with a very high number of mosquitoes (> 24,974) collected in two sites (Etsha 1 and Kandalangondi) and a much lower number of mosquitoes (< 1,500) trapped in the other two sites (Etsha 6 and Gumare). We hypothesized that these local differences were probably due to the distance between the trapping sites and the edge of the Delta. Indeed, Kandalangondi and Etsha 1 being closer to the Delta they benefit from higher and more regular water availability (Figs. 1 and 4). It was worth noting that previous studies on the spatial distribution of *Cx. pipiens* in Europe showed weak correlations between land cover and *Cx. pipiens* abundance [55, 56], stressing the ability of this species to colonize diverse breeding sites. Our results suggest that it might not be the case in constrained environments, where water presence determines the availability of mosquito breeding sites. Indeed, predicted and observed abundance values poorly fitted under scenario 1 (temperature alone) although the fit was better under all other scenarios. Both, the presence of permanent Delta waters and seasonal regime of rainfall, were likely to influence the population dynamics of this species by affecting (i) the environmental carrying capacity of larvae and pupae, and (ii) the adult mortality rate related to seeking behaviour. The efficiency of scenario 4 (temperature, rainfall and flooding), for which the correlation coefficient between observed and predicted values was the highest, suggests a strong influence of both flooding and rainfall on *Cx. pipiens* dynamics in the Okavango Delta area. The impact of flooding is expected to be higher for the sites close to the Delta River and the impact of rainfall higher for the sites located further from the Okavango River. Our results confirmed these trends, showing a better fit between predicted and observed values obtained in Scenario 2 (temperature and rainfall amounts) than in Scenario 3 (temperature and flooding) for Kandalangondi and Gumare sites, both located further south from Okavango River. Moreover, taking into account local flooding seemed to explain the spatial variability in catches observed among the four trapping sites (Fig. 6) and supports the hypothesis that local water variations have an important impact on *Cx. pipiens* dynamics and abundance. To test this hypothesis in the field, measurements of water variations could be conducted around each study site. Additional local measures of meteorological conditions should also be conducted around each site to confirm the hypothesis that there is a low variability in temperature among the study sites.

The sensitivity analysis allowed the identification of the key parameters driving *Cx. pipiens* population dynamics in the Delta (Fig. 7). As for other species, an improved knowledge of those parameters through laboratory or field studies would be required to increase the precision of the model predictions [42]. Moreover, those parameters allow targeting potential control points in the biological system which can be used to discuss possible scenarios of vector control strategies [57]. Key parameters are dependent on the species but also on the environment, for instance, in Osijek, Croatia (see Loncaric et al*.* [57]) highlighted the pupae and adults as sensitive stages of *Cx. pipiens* life-cycle. In our study area, control strategies focused on adult stages are likely to have a bigger effect than those targeting aquatic stages (pupa or eggs).

The increased abundance of *Cx. pipiens* simulated in December was consistent with the higher seroprevalences of RVFV in human samples collected during the same month in another area of Northern Botswana observed by Tessier et al*.* [58]. In other words, the increase of transmission rate in humans in Nov-Dec-Jan suggested by Teissier et al*.* [58] could be explained by an increased *Cx. pipiens* abundance. The relationships between mosquito-borne disease transmission and vector dynamics have commonly been established [59–62]. Nevertheless, no RVFV could be detected despite processing 65 % of the mosquito catches and using highly sensitive methods for the detection of arboviruses. Analysis concentrated on those vector species known to play a role in the epidemiology of the diseases elsewhere. This absence of virus in mosquitoes was not surprising according to the absence of active circulation of virus. Indeed, in our study area, no clinical outbreaks have ever been detected despite the reported presence of antibodies in humans, livestock and free ranging African buffalo [11, 58]. Under those circumstances, the probability of detecting RVFV in mosquitoes in the inter-epidemic period is extremely low [63]. Moreover, since the detected antibodies were only indicative of past and not recent infections, further epidemiological surveys in human and animal patients using serological tests able to detect recent infections (IgM antibodies) are necessary. In Southern Africa, RVF is more commonly controlled by vaccination of animal populations rather than through vector control. However, our results could be useful to understand patterns of *Cx. pipiens* dynamics and the vector control efforts should be focused on adult stages rather than aquatic stages. In the case of an emergence of a RVF outbreak, public health agencies could use the temporal and spatial predictions of mosquito abundance provided by our study based on real-time collected meteorological data to set up adapted surveillance and control plans. Agreeing to previous studies, extreme weather events might create the necessary conditions for vector borne diseases such as RVF, to expand beyond its geographical area, triggering unexpected impacts on the animal and human health of newly affected countries [64–66].

The population dynamics model used in this study has been implemented so far, in three different and very diverse ecological contexts: (i) the French Camargue region to analyse *Anopheles*, *Culex* and *Aedes* mosquito population dynamics in a temperate wetland [18, 67]; (ii) the French coastal region of Cote d’Azur to study *Aedes* mosquito population dynamics in an urban area [42]; and (iii) our study, where *Cx. pipiens* population dynamics was described in a tropical wetland environment. This study verified versatility of the model and suggested that it could possibly be used in a wider range of applications. The model predictability under conditions of inter-annual variability had not been assessed and could be explored by setting up mosquito trapping campaigns through several years or at least each month during one complete year. To improve the model predictions and better explain the observed spatial variability in the vector abundance, information on land cover and vegetation could be accounted for in the model, provided that land cover maps are available on the study area, or derived from satellite imagery [68, 69]. Moreover, to assess the response of the model for the entire life-cycle, additional sampling efforts catching different mosquito life stages (adult/eggs/larvae/pupae) with a higher sampling frequency are required. Finally, host density (cattle, wildlife) may also be an important factor to be taken into account, because of its potential impact on vector densities.

The vector dynamics simulated by our model could be used as inputs for a compartmental (SIR) model [70], other transmission models [71, 72]. Our model could also be coupled to predictive climate models such as proposed by Guis et al*.* (2011) [73]. For instance, the three identified climatic factors were relevant to predict vector population increase and could provide the first step for establishing risk maps of RVF emergence or entomological risk, taking into account the seasonal variations of host and vector distributions, and allowing the establishment of assumptions about the effect of the environmental factors on RVF transmission. This kind of application could bring better knowledge but also enable testing and improving of control strategies (vaccinations or vector-control strategies [74]).

## Conclusions

In this paper, we provide original data describing, for the first time, the diversity of mosquito populations in the region of Okavango Delta in northern Botswana where RVFV is suspected to circulate without reports of clinical outbreaks. Our work also highlights a large overabundance of *Cx. pipiens* compared to other mosquito species in the most populated areas of the western side of the Okavango Delta. The rare occurrence of *Aedes* spp. could be explained by an unfavourable environment (permanent water) avoiding the desiccation of *Aedes* spp. eggs required for their hatching. However, our results should be considered with caution, since potential biases could have occurred due to the mosquito capture design adopted. Further sampling during the dry season and over several years is strongly recommended.

The mosquito population model developed and validated by our field trapping data was able to reproduce *Cx. pipiens* abundance and dynamics using three environmental and meteorological inputs (temperature, rainfall and proportion of flooded surface). It highlights the significant role of permanent water in this particular region and the relative influence of both rainfall and flooded surface at local level depending on the distance to the banks of the Delta. To the best of our knowledge, it was also the first time that the population dynamics of *Cx. pipiens* in a semi-arid environment was modelled using a mechanistic approach. Our model allowed testing different assumptions on the main drivers of *Cx. pipiens* dynamics in the Okavango region and, despite the low number of catching episodes, it accurately predicted the spatial variability of *Cx. pipiens* abundance on the western side of the Delta and the major trends in the annual fluctuations of host-seeking adults in the two trapping sites closer to the Okavango River which are probably more strongly influenced by flooding.

Our study is a necessary first step in the understanding the potential dynamics of RVF in the Okavango Delta. However, increased knowledge on RVFV epidemiology requires additional field data from exposed human and animal populations, complementary vector sampling and continued development of modelling techniques for exploring plausible disease transmission scenarios, emergence mechanisms and the potential impact of intervention strategies.

## Abbreviations

asl, above sea level; MODIS, moderate resolution imaging apectroradiometer; ODE, ordinary differential equations; RVF, Rift Valley fever; RVFV, Rift Valley fever virus; TRMM, tropical rainfall measuring.

## Additional files

Additional file 1: Serological analysis (Sampling strategy, laboratory analysis and results). (PDF 71 kb) Additional file 2: Mapping flooding extent method. Figure in Additional file 2. Maps of Modified Normalized Difference Water Index (MNDWI) derived from MODIS imagery at different dates corresponding to the study period. (PDF 3185 kb) Additional file 3 Detail of the ordinary differential equation system. (PDF 139 kb)
