# Supplementary material for: Rift Valley fever vector diversity and impact of meteorological and environmental factors on Culex pipiens dynamics in the Okavango Delta, Botswana
Source: Parasit Vectors. 2016 Aug 8;9:434. doi: 10.1186/s13071-016-1712-1 (PMC4977755; doi:10.1186/s13071-016-1712-1)
Supplement: Additional file 1: — Serological analysis (Sampling strategy, laboratory analysis and results). (PDF 71 kb) [file 13071_2016_1712_MOESM1_ESM.pdf]

Additional files

Additional file 1.

Serological analysis (Sampling strategy, laboratory analysis and results). Ten crushpens were initially chosen randomly out of a list of 42 crushpens located adjacent to the game fence, which forms the physical boundary between the Okavango delta and the communal livestock production areas. The final list of 10 crushpens was compiled according to their proximity to the fence and the availability of the National Veterinary Services teams to operate in the area between the 30th April and the 3rd May 2010, during the routine Foot and Mouth Disease vaccination campaign. In every crushpen, a total of 50 cattle belonging to 10 different herds were randomly chosen (5 cattle per herd). Therefore, a total of 100 samples per day were taken (50 cattle/crushpen and two crushpens per day). Samples were collected during 5 days between the 28th of April and the 3rd of May.

The samples were sent to the Virology Laboratory of ARC-OVI in Ondersterpoort, South AFrica for the detection of IgG antibodies with an in-house Indirect ELISA test. A serum sample was considered positive if it had a titer equal or higher to a serum dilution of 1:10.

The results per crush are specified in Table 1. In our sample, 5 out of 10 crushpens showed detectable IgG antibodies at prevalences ranging between 2% and 11.6%. The overall prevalence among the 481 animal tested was 3% [1.7%-5.1%]. This sampling exercise is part of a larger survey performed in buffalo and cattle in Northern Botswana [1]. The same sera were also tested at the NICD, reference laboratory for RVF in Southern Africa, to detect neutralizing antibodies with the Viral Neutralization Test (VNT), which is considered as the gold standard for serological detection of RVF antibodies. Its analysis provided additional and irrevocable evidence that the RVF virus is widely circulating in buffalo and cattle from Northern Botswana.

Références

1. Jori F, Alexander KA, Mokopasetso M, Munstermann S, Moagabo K, Paweska JT. Serological evidence of Rift Valley fever virus circulation in domestic cattle and African buffalo in Northern Botswana (2010-2011). Frontiers in Veterinary Science. Accepted in 2015 Oct;.
