# Supplementary material for: Rift Valley fever vector diversity and impact of meteorological and environmental factors on Culex pipiens dynamics in the Okavango Delta, Botswana
Source: Parasit Vectors. 2016 Aug 8;9:434. doi: 10.1186/s13071-016-1712-1 (PMC4977755; doi:10.1186/s13071-016-1712-1)
Supplement: Additional file 2: — Mapping flooding extent method. Figure in Additional file 2. Maps of Modified Normalized Difference Water Index (MNDWI) derived from MODIS imagery at different dates corresponding to the study period. (PDF 3185 kb) [file 13071_2016_1712_MOESM2_ESM.pdf]

Additional files

Additional file 2.

Mapping flooding extent method. Moderate Resolution Imaging Spectroradiometer (MODIS) satellite images were acquired from Land Process Distributed Active Archive Center (https://lpdaac/usgs/gov/). MODIS Terra 8-day composite images of surface reflectance estimates at 500 m spatial resolution (product MOD09A1) covering the Okavango Delta were collected between 2005 and 12. Preprocessing steps consist in masking the low quality pixels and the pixels covered by clouds using the surface reflectance quality file, and performing a linear temporal interpolation of the masked pixels using the ENVI IDL software 4.8 [1]. Temporal series of water indices were then computed from the reflectance values of the cleaned images according to the following equation: MNDWI= $\frac{(G-SWIR)}{G+SWIR}$), with G: Reflectance in green wavelength; SWIR: reflectance in short wave infrared range. A threshold was then applied on MNDWI images to delineate free water (MNDWI threshold value=-0.4). Image processing was performed using the ENVI IDL software 4.8 [1]. GIS analyses were performed using ArcGIS 10.1 + Spatial analyst extension [2].

Références

1. Exelis V. IDL software, Boulder, CO, USA; 2014.
2. McCoy J, Johnston K, systems research institute E. Using ArcGIS spatial analyst: GIS by ESRI. Environmental Systems Research Institute; 2001.

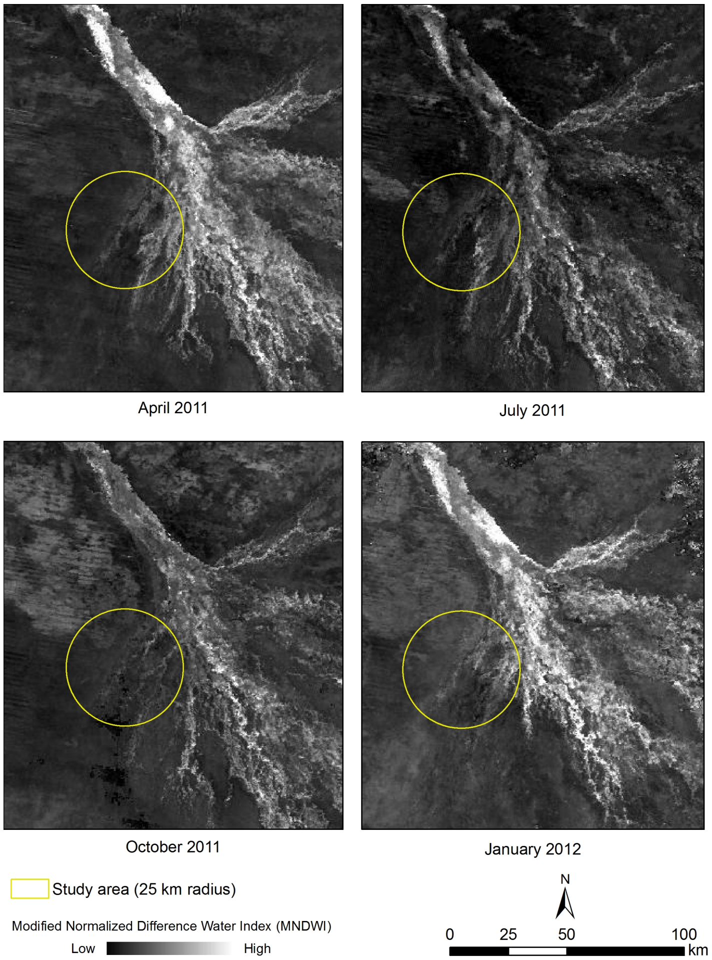

Maps of Modified Normalized Difference Water Index (MNDWI) derived from MODIS imagery at different dates corresponding to the study period
