# Supplementary material for: Rift Valley fever vector diversity and impact of meteorological and environmental factors on Culex pipiens dynamics in the Okavango Delta, Botswana
Source: Parasit Vectors. 2016 Aug 8;9:434. doi: 10.1186/s13071-016-1712-1 (PMC4977755; doi:10.1186/s13071-016-1712-1)
Supplement: Additional file 3 — Detail of the ordinary differential equation system. (PDF 139 kb) [file 13071_2016_1712_MOESM3_ESM.pdf]

Parasites &Vectors

Additional files

Additional file 3.

Detail of the ordinary differential equation system

$$
\left\{
\begin{array}{rcl}
\dot{E} &=& \gamma_{Ao}(\beta_1 A_{1o} + \beta_2 A_{2o}) - (\mu_E + f_E)E \\
\dot{L} &=& f_E E - [m_L(1 + \frac{L}{k_L}) + f_L]L \\
\dot{P} &=& f_P P - [m_P + f_P]P \\
\dot{A}_{em} &=& f_P P \sigma e^{[-\mu_{em}(1+\frac{P}{k_P})]} - [z \times m_A + (1-z) \times \mu_A + z \times \gamma_{Aem}]A_{em} \\
\dot{A}_{1h} &=& z \times \gamma_{Aem} A_{em} - (m_A + m_r + \gamma_{Ah})A_{1h} \\
\dot{A}_{1g} &=& \gamma_{Ah} A_{1h} - (m_A + f_{Ag})A_{1h} \\
\dot{A}_{1o} &=& \gamma_{Ag} A_{1g} - (m_A + m_r + \gamma_{Ao})A_{1o} \\
\dot{A}_{2h} &=& \gamma_{Ao}(A_{1o} + A_{2o}) - (m_A + m_r + \gamma_{Ah})A_{2h} \\
\dot{A}_{2g} &=& \gamma_{Ah} A_{2h} - (m_A + f_{Ag})A_{2g} \\
\dot{A}_{2o} &=& f_{Ag} A_{2g} - (m_A + m_r + \gamma_{Ao})A_{2o}
\end{array}
\right.
\quad , with\ z = \left\{ \begin{array}{l} 0\ during\ diapause \\ 1\ otherwise \end{array} \right.
$$

Model parameters are in Greek letters. They are constant. For stage X, γX is the transition rate to the next stage, βX the egg laying rate and μX the mortality rate. Finally, σ is the sex-ratio at the emergence, and only the female proportion of emerging pupae that survives to emergence is considered in the stage “emerging adults”.

Model functions are in Latin letters. They depend on parameters and are climate-driven functions (i.e. functions involving temperature, flooding extent and precipitation varying over time). For stage X, fX is the transition function to the next stage, mX the mortality function and kX the environment carrying capacity which limits also the population growth due to density-dependent. The density-dependent survival rate is expressed as a probability. We also assumed an additional adult mortality rate related to the seeking behaviour, mr, which is applied only on adult stages involving risky movements. This rate includes mortality related to host seeking and oviposition site seeking behaviours.
